# A Single-Step Sequencing Method for the Identification of *Mycobacterium tuberculosis* Complex Species

**DOI:** 10.1371/journal.pntd.0000253

**Published:** 2008-06-18

**Authors:** Zoheira Djelouadji, Didier Raoult, Mamadou Daffé, Michel Drancourt

**Affiliations:** 1 Unité des Rickettsies CNRS UMR6020, IFR 48, Faculté de Médecine, Université de la Méditerranée, Marseille, France; 2 Département de Mécanismes Moléculaires des Infections Mycobactériennes, Institut de Pharmacologie et Biologie structurale, Toulouse, France; University of Tennessee, United States of America

## Abstract

**Background:**

The *Mycobacterium tuberculosis* complex (MTC) comprises closely related species responsible for strictly human and zoonotic tuberculosis. Accurate species determination is useful for the identification of outbreaks and epidemiological links. *Mycobacterium africanum* and *Mycobacterium canettii* are typically restricted to Africa and *M. bovis* is a re-emerging pathogen. Identification of these species is difficult and expensive.

**Methodology/Principal Findings:**

The Exact Tandem Repeat D (ETR-D; alias Mycobacterial Interspersed Repetitive Unit 4) was sequenced in MTC species type strains and 110 clinical isolates, in parallel to reference polyphasic identification based on phenotype profiling and sequencing of *pncA*, *oxyR*, *hsp65*, *gyrB* genes and the major polymorphism tandem repeat. Inclusion of *M. tuberculosis* isolates in the expanding, antibiotic-resistant Beijing clone was determined by Rv0927c gene sequencing. The ETR-D (780-bp) sequence unambiguously identified MTC species type strain except *M. pinnipedii* and *M. microti* thanks to six single nucleotide polymorphisms, variable numbers (1–7 copies) of the tandem repeat and two deletions/insertions. The ETR-D sequencing agreed with phenotypic identification in 107/110 clinical isolates and with reference polyphasic molecular identification in all isolates, comprising 98 *M. tuberculosis*, 5 *M. bovis* BCG type, 5 *M. canettii*, and 2 *M. africanum*. For *M. tuberculosis* isolates, the ETR-D sequence was not significantly associated with the Beijing clone.

**Conclusions/Significance:**

ETR-D sequencing allowed accurate, single-step identification of the MTC at the species level. It circumvented the current expensive, time-consuming polyphasic approach. It could be used to depict epidemiology of zoonotic and human tuberculosis, especially in African countries where several MTC species are emerging.

## Author Summary

The *Mycobacterium tuberculosis* complex (MTC) comprises several closely related species responsible for strictly human and zoonotic tuberculosis. Some of the species are restricted to Africa and were responsible for the high prevalence of tuberculosis. However, their identification at species level is difficult and expansive. Accurate species identification of all members is warranted in order to distinguish between strict human and zoonotic tuberculosis, to trace source exposure during epidemiological studies, and for the appropriate treatment of patients. In this paper, the Exact Tandem Repeat D (ETR-D) intergenic region was investigated in order to distinguish MTC species. The ETR-D sequencing unambiguously identified MTC species type strain except *M. pinnipedii* and *M. microti*, and the results agreed with phenotypic and molecular identification. This finding offers a new tool for the rapid and accurate identification of MTC species in a single sequencing reaction, replacing the current time-consuming polyphasic approach. Its use could assist public health interventions and aid in the control of zoonotic transmission in African countries, and could be of particular interest with the current emergence of multidrug-resistant and extended-resistance isolates.

## Introduction

**Figure 1:**
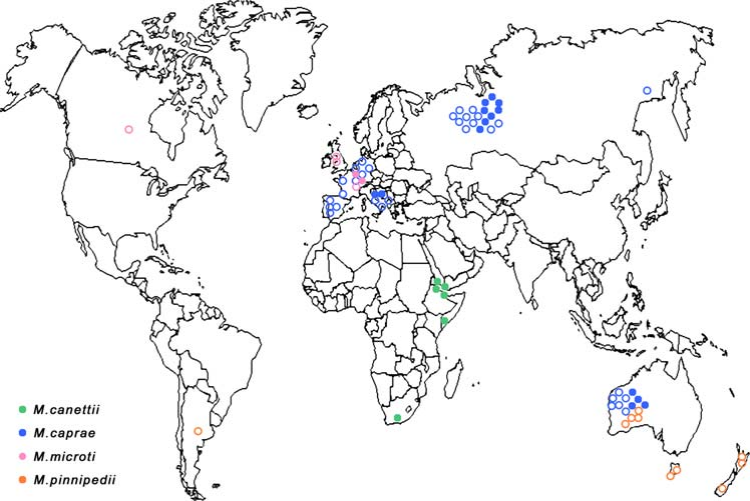
Distribution of rare *Mycobacterium tuberculosis* complex species in humans (filled circles) and animals (open circles). Green circles, *M. canettii*; blue circles, *M. caprae*; pink circles, *M. microti*; orange circles, *M. pinnipedii*.

**Figure 2:**
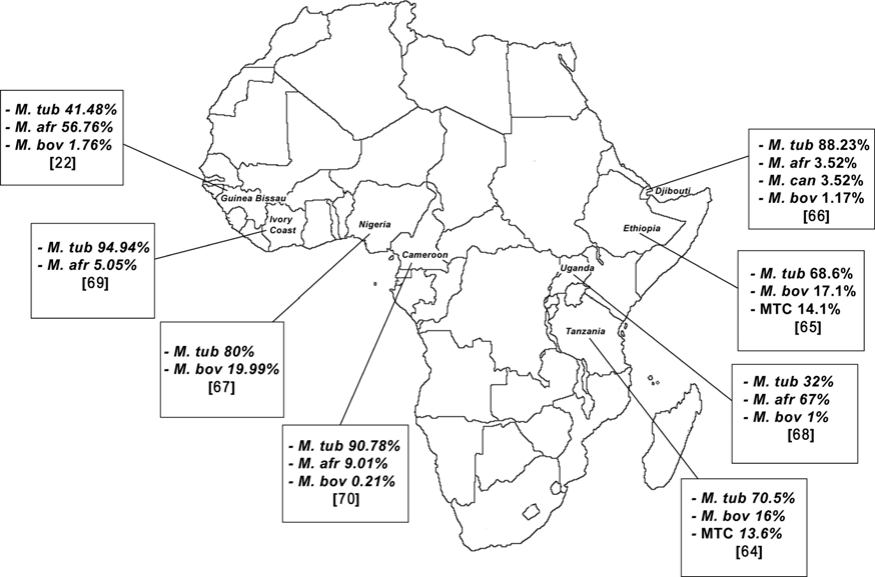
Distribution of *Mycobacterium tuberculosis* complex species in human tuberculosis cases in Africa. Based on a review of papers published in 1999–2007 [22], [64]–[70]. For each country, prevalence rates and reference are indicated in cartoons. Mtub, *M. tuberculosis*; M afr, *M. africanum*; Mbov, *M. bovis*; Mcan, *M. canettii*; MTC, *Mycobacterium tuberculosis* complex.

The *Mycobacterium tuberculosis* complex (MTC) comprises several closely related species responsible for strictly human and zoonotic tuberculosis (Figure 1). In addition to *M. tuberculosis*, which represents the leading cause of human tuberculosis worldwide and is now emerging as extensively drug-resistant tuberculosis strains [1], other MTC species have been found in patients, typically in African countries (Figure 2). *Mycobacterium bovis* is a re-emerging, zoonotic agent of bovine tuberculosis [2] whose prevalence probably depends on variations in direct exposure to cattle and consumption of unpasteurised dairy products [3]. The prevalence of *Mycobacterium africanum* type I (West Africa) and type II (East Africa) [4] has decreased in several African countries over the last decades [5],[6]. *Mycobacterium canettii*, a rare MTC species, has been isolated recently in patients exposed in Africa [7]. *Mycobacterium microti*, a vole and small rodent pathogen [8] that is closely related to the so-called Dassie-bacillus and infects small mammals in South Africa and the Middle East [9],[10], has been isolated in humans [11]. *Mycobacterium caprae* is a rare cause of tuberculosis in cattle [12],[13] and zoonotic tuberculosis in humans [14] while *Mycobacterium pinnipedii* has been isolated from seal lions and fur seals [15]. A recent description of the re-emergence of *M. bovis* in cattle, along with the direct interhuman transmission of this zoonotic organism [16] in a six-case cluster that included one death in United Kingdom [17], illustrates the potential of emerging and re-emerging zoonotic tuberculosis due to MTC species other than *M. tuberculosis* and the necessity for accurate species identification.

Accurate species identification of all MTC members is warranted in order to distinguish between strict human and zoonotic tuberculosis and to trace source exposure during epidemiological studies. Indeed, phenotypic methods of identification relying on colony morphology, oxygen preference, niacin accumulation, nitrate reductase activity, growth kinetics and resistance to thiophene-2-carboxylic acid hydrazide (TCH) and PZA [18] are hampered by slow growth of MTC members and subjective interpretation of colony morphology and cross-resistance to drugs [19]. They do not always allow unambiguous species identification in every case. Recent studies of MTC species responsible for animal and human tuberculosis in tropical countries have relied on molecular methods including mycobacterial interspersed repetitive-unit-variable-number tandem-repeat (MIRU-VNTR) typing, IS*6110*-RFLP and spoligotyping [20]–[22]. Molecular differentiation of MTC members has been complicated by low sequence variability at the nucleotide level, illustrated by a 85–100% DNA/DNA relatedness and a 99–100% 16S rDNA sequence similarity [23],[24]. Nucleic acid-based assays such as acridinium ester-labelled DNA probes (AccuProbe; Gene Probe Inc, San Diego, CA) have proven to be reliable tools for assigning an isolate to the MTC [25],[26], but they do not allow for identification at the species level. Molecular identification based on deleted regions (RD), RD1, RD9 and RD10 [27], are limited by the necessity of interpreting negative results in the case of the absence of a specific deletion. The detection of single nucleotide polymorphisms (SNP) in the *pncA* gene [28], the *oxyR* locus [29], the *mtp40* gene [30], and the restriction fragment length polymorphism of the *hupB* gene [31] differentiated *M. tuberculosis* from *M. bovis* but not from other MTC species. The major polymorphism of tandem repeat (MPTR) sequencing differentiated *M. tuberculosis* (Sequevar long), *M. bovis* and *M. microti* (Sequevar Med-G), *M. bovis* BCG (Sequevar Med-C) and *M. africanum* (Sequevar short), but other MTC species were not studied [32]. The *gyrB* gene proved to be an effective target [33],[34], as an identification scheme has been proposed based on Pyrosequencing analysis of four single nucleotide polymorphism (SNPs) in *gyrB* [35], and a DNA strip based on *gyrB* is commercially available (HAIN Genotype MTBC DNAstrip test, Hain Lifescience, Nehren, Germany) [36]. Both approaches, however, fail to differentiate *M. tuberculosis* from *M. africanum* type II and *M. canettii;* and *M. africanum* type I from *M. pinnipedii*. IS*6110*-RFLP, VNTR typing and Spoligotyping [22],[37] emerged as reference methods to study the diversity of MTC species in resource-limited countries, despite the fact that these methods may not recognize rarely encountered species and may not appreciate the entire genetic diversity of strains, as they are not based upon the sequencing of molecular targets [38].

When investigating intergenic spacers in the genotyping of *M. tuberculosis*, we found that one spacer, previously identified as the Exact Tandem Repeat D (ETR-D) [39] and aliased Mycobacterial Interspersed Repeat Unit 04 (MIRU04) [40], exhibited a variable sequence among *M. tuberculosis* isolates. Analysis of this spacer had been previously shown to distinguish between *M. bovis* and the *M. bovis* BCG type [41]. We therefore further investigated whether sequencing the ETR-D could identify all of the MTC at the species level. In this study, we demonstrate that ETR-D sequencing offers a new tool for the rapid and accurate identification of MTC species in a single reaction.

## Methods

### Bacterial isolates

*M. tuberculosis* CIP103471, *M. bovis* CIP105050, *M. africanum* CIP105147^T^ (type I), *M. bovis* BCG vaccine strain type 105060, *M. microti* CIP104256^T^, *M. canettii* CIP140060001^T^, *M. pinnipedii* ATCC BAA-688, and *M. caprae* CIP105776^T^ reference strains were purchased from the Collection Institut Pasteur (CIP, Paris, France) and American Type Culture Collection (ATCC, Rockville, USA). The following non-tuberculosis mycobacteria were tested in order to assess the specificity of ETR-D spacer sequencing: *Mycobacterium avium* IWGMT49^ T^, *Mycobacterium intracellulare* CIP104243^ T^, *Mycobacterium chimaera* CIP107892^ T^, *Mycobacterium colombiense* CIP108962^ T^, *Mycobacterium haemophilum* CIP105049^ T^, *Mycobacterium ulcerans* CIP105425^ T^, *Mycobacterium xenopi* CIP104035^ T^, *Mycobacterium abscessus* CIP104536^ T^, *Mycobacterium chelonae* CIP104535^ T^, *Mycobacterium fortuitum* ATCC49404 and *Mycobacterium mucogenicum* CIP 105223^ T^. Quality of DNA was controlled by parallel partial *rpoB* PCR amplification as previously described [42]. One hundred and ten MTC clinical isolates (Table 1) recovered from Microbiology Laboratory in Marseille (n = 102), from Institut Pasteur in Djibouti (n = 3) and from Institut de Pharmacologie et Biologie Structurale, Toulouse (n = 5) were also analyzed. All isolates were identified as members of the MTC by phenotypic characterization and a gene probe assay according to the manufacturer (AccuProbe; Gene Probe Inc, San Diego, Calif). This study was approved by the ethics committee of the Institut Féfératif de Recherche 48, Marseilles, France.

**Table 1:** Origin of *Mycobacterium tuberculosis* complex reference strains and clinical isolates analyzed in this study

Isolates	Origin
*M. tuberculosis*	CIP103471
*M. africanum*	CIP105147^T^
*M. bovis*	CIP105050
*M. bovis* BCG type	Vaccine strain105060
*M. caprae*	CIP105776^T^
*M. canettii*	CIP140060001^T^
*M. microti*	CIP104256^T^
*M. pinnipedii*	ATCC BAA-688
Clinical isolates	
n = 57	Sputum
n = 48	Lymph node
n = 3	Pleural liquid
n = 2	Abscess

T : type strain.

### Phenotypic identification

Phenotypic characterisation included colony morphology, a urease test controlled after 3 and 18 hour incubation, and oxygen consumption measured after inoculation of a 0.2 ml actively growing mycobacterial suspension into 40 ml of Middlebrook 7H10 into the Bactec 9000MB system (Becton and Dickinson, Le Pont de la Claix, France) after a 3-week incubation. Drug susceptibility tests for thiophene-2-carboxylic acid hydrazide (TCH) and PZA were performed as previously described [43].

### Reference tests for molecular identification

The identification of reference strains and clinical isolates identified as *M. bovis* BCG type, *M. canettii* and *M. africanum* by ETR-D sequencing (see below) was confirmed by parallel reference molecular tests. Every isolate coated on beads was inactivated as previously described [44] and the DNA was extracted using a Qiagen kit (Qiagen, Courtaboeuf, France). DNA was used as a template for PCR amplification of *pncA, oxyR*, *hsp65*, *gyrB* genes and sequence analysis of MPTR was performed as previously described [28], [29], [32]–[34],[45] In addition, we sequenced the Rv0927c-pstS3 intergenic region in all clinical isolates identified as *M. tuberculosis* in order to identify the Beijing genotype [46]. Amplified products were visualized by agarose gel electrophoresis and direct sequencing was performed as described above. Sequences were edited using the Auto assembler program (Applied Biosystems, Courtaboeuf, France) and aligned using CLUSTAL W (http://pbil.ibcp.fr). Original sequences were deposited into GenBank (http://www.ncbi.nlm.nih.gov/sites/entrez/).

### ETR-D spacer sequencing

Amplification and sequencing of the ETR-D spacer located between the putative histidine kinase *Senx3* upstream and the sensory transduction protein *Regx3* downstream were done using direct primers: 5′-GTTGATCGAGGCCTATCACG-3′ and 5′-GAATAGGGCTTGGTCACGTA-3′. The PCR mixture contained 33 µl H_2_O, 5 µl 10× buffer (Qiagen), 2 µl 25× MgCl_2_, 5 µl 10× dDNTP, 1 µl forward primer, 1 µl reverse primer, 0.25 µl hotstart Taq (Qiagen) and 2 µl target DNA. Appropriate negative controls consisting of PCR mix without target DNA were also included. PCRs were performed using the following program: 15 min enzyme activation at 95°C, followed by 34 cycles consisting of 95°C for 30 s, 58°C for 30 s, 72°C for 1 min, followed by a 5 min elongation step at 72°C. After agarose gel electrophoresis, PCR products were purified and subjected to sequencing in both directions by using the BigDye Terminator 1.1 Cycle Sequencing kit (Applied Biosystems). Sequencing electrophoresis was performed on a 3130 genetic analyzer (Applied Biosystems). The sequences were edited using the Auto assembler program (Applied Biosystems) and aligned using CLUSTAL W (http://pbil.ibcp.fr). Original ETR-D sequences were deposited into Genbank (http://www.ncbi.nlm.nih.gov/sites/entrez/).

## Results

### Phenotypic identification (Table 2)

As for reference strains, *M. tuberculosis* exhibited eugonic growth that was inhibited by the presence of PZA but not by TCH and showed aerophilic growth on Middlebrook agar positive urease at 18 hours. *M. bovis* and *M. bovis* BCG type strains exhibited microaerophilic dysgonic growth, did not grow in the presence of TCH, but were resistant to PZA and exhibited a positive urease activity at 3 hours for *M. bovis* BCG type and at 18 hours for *M. bovis*. *M. africanum* type I differed from *M. bovis* by its susceptibility to PZA. *M. canettii* exhibited eugonic growth in the presence of PZA and TCH, and showed a positive urease activity at 3 hours and aerophilic growth on Middlebrook. *M. microti*, *M. capare* and *M. pinnipedii* exhibited eugonic growth that was inhibited by TCH and PZA, and a positive urease at 18 hours. As for clinical isolates, 101/110 of isolates were phenotypically identified as *M. tuberculosis,* 5 as *M. canettii*, 3 as *M. bovis* BCG type and one as *M. africanum*.

**Table 2:** Biochemical and antibiotic susceptibility profiles observed for *M. tuberculosis* complex reference strains and clinical isolates in this study

Isolates	Colony morphology	Urease 3 hours	test 18 hours	PZA sensitivity	TCH sensitivity	Oxygenpreference
*M. tuberculosis* CIP103471	Eugonic	-	+	S	R	Aerophilic
*M. bovis* CIP105050	Dysgonic	-	+	R	S	Microaerophilic
*M. bovis* BCG type 105060	Dysgonic	+	+	R	S	Microaerophilic
*M. africanum* T CIP105147	Dysgonic	-	+	S	S	Microaerophilic
*M. canettii* T CIP140060001	Eugonic	+	+	R	R	Aerophilic
*M. caprae* T CIP105776	Eugonic	-	+	S	S	Aerophilic
*M. microti* T CIP104256	Eugonic	-	+	S	S	Aerophilic
*M. pinnipedii* ATCC BAA-688	Eugonic	-	+	S	S	Aerophilic
Clinical isolates						
101	Eugonic	-	+	S	R	Aerophilic
1	Dysgonic	-	+	S	S	Microaerophilic
3	Dysgonic	+	+	R	S	Microaerophilic
5	Eugonic	+	+	R	R	Aerophilic

T : type strain, +: positive results, -: negative results, R: drug resistant, S: drug susceptible, TCH: thiophene-2-carboxylic acid hydrazide (5 µg/ml), PZA: pyrazinamide (50 µg/ml).

### Reference molecular identification (Table 3)

In all PCR experiments, negative controls remained negative. All reference strains and clinical isolates yielded an amplicon of the expected size when amplified for *pnc*A, *oxyR*, *hsp65*, *gyrB* genes, Rv0927c-pstS3 intergenic region and MPTR. By comparison with *M. tuberculosis*, the 410-bp *oxyR* gene sequence exhibited a previously known A_285_G polymorphism in *M. bovis* and *M. bovis* BCG type [29] and a newly identified T_136_G polymorphism in *M. canettii*. The 561-bp *pnc*A gene sequence exhibited a previously known G_253_C polymorphism in *M. bovis* and *M. bovis* BCG type [28] and a G_222_A polymorphism in *M. canettii* [47]. The 441-bp *hsp65* gene exhibited a previously known T_235_C polymorphism in *M. canettii* [45] and a newly identified G_376_C polymorphism in *M. africanum* type I. The 1.020-bp *gyrB* gene sequence exhibited an identical sequence in *M. tuberculosis, M. canettii* and *M. caprae*, a previously known A_756_G polymorphism in *M. bovis* and *M. bovis* BCG type, a T_675_C polymorphism in *M. microti*, and an identical sequence was identified in common with *M. africanum* type I and *M. pinnipedii* [34]. Sequence analysis of MPTR (300-bp) exhibited a unique sequence for *M. tuberculosis* (Sequevar Long), *M. africanum* type I strain (Sequevar Short), *M*. *bovis* and *M. microti* (Sequevar MED-G) and *M. bovis* BCG type (Sequevar MED-C) [32]; MPTR sequencing newly identified C_99_T, G_164_C and A_267_G polymorphisms in *M. canettii*; the *M. caprae* strain exhibited Sequevar Long in common with *M. pinnipedii* and *M. tuberculosis* reference strains. Original sequences found in this study were deposited in GenBank under the following accession numbers (GenBank: EF656461, EF 656463, EF 656464).

**Table 3:** Single nucleotide polymorphisms in five housekeeping genes and MPTR sequence analysis in eight MTC reference strains

Strains	*oxyR*		*pnc*A		*hsp*65		*gyrB*			MPTR			
	136	285	222	253	235	376	675	756	Sequence	99	164	267	Sequence
*M. tuberculosis* CIP103471	G	G	A	C	C	C	C	G		T	C	G	Long
*M. africanum* T CIP105147	G	G	A	C	C	G*	C	G	sequevar	T	C	G	Short
*M. bovis* BCG type105060	G	A	A	G	C	C	C	A		T	C	G	MED-G
*M. bovis* CIP105050	G	A	A	G	C	C	C	A		T	C	G	MED-G
*M. canettii* T CIP140060001	T*	G	G	C	T	C	C	G		C*	G*	A*	Long
*M. microti* T CIP104256	G	G	A	C	C	C	T	G	sequevar	T	C	G	MED-G
*M. pinnipedii* ATCC 688	G	G	A	C	C	C	C	G	sequevar	T	C	G	Long
*M. caprae* T CIP105776	G	G	A	C	C	C	C	G		T	C	G	Long

T : type strain,

*: : Polymorphism identified newly in this study.

Sequence analysis of clinical isolates using the five previous targets yielded four different profiles. One profile comprised 98 isolates identified as *M. tuberculosis*, including three isolates identified as W-Beijing strains using a G_127_A polymorphism in Rv0927c-pstS3 intergenic region, a second profile comprised five isolates identified as *M. bovis* BCG type; a third profile included five isolates identified as *M*. *canettii* and a fourth profile included two isolates identified as *M. africanum* type I.

### ETR-D sequence analysis

For all ETR-D experiments, negative controls remained negative. All the non-tuberculosis mycobacteria yielded a negative ETR-D PCR amplification whereas they yielded an amplicon of the expected size through *rpo*B PCR amplification. The size of PCR products obtained with MTC reference strains varied from 497-bp for *M. canettii*, 545-bp for *M. bovis*, 564-bp for *M. caprae*, 598-bp for *M. bovis* BCG type, 651-bp for *M. africanum* type I, 805-bp for *M. tuberculosis* and 959-bp for *M. microti* and *M. pinnipedii*. Each of the eight reference strains exhibited a unique ETR-D sequence, exhibiting one to seven copies of a tandem repeat, six mutations and two deletions/insertions.

*M. tuberculosis* exhibited three different alleles combining two or five copies of a 77-bp repeat unit and one T/G SNP at the fifth base of the tandem repeat, in addition to one 53-bp repeat unit. The *M. tuberculosis* reference strain exhibited five copies of a 77-bp repeat unit followed by one 53-bp repeat unit copy; *M. microti* and *M. pinnipedii* exhibited seven copies of a 77-bp repeat unit and one 53-bp repeat unit, *M. africanum* type I exhibited three copies of a 77-bp repeat unit and one 53-bp repeat unit, in addition to one T/C polymorphism at position 75. *M. bovis* exhibited four copies of a 77-bp repeat unit and one 53-bp repeat unit in addition to an A/G SNP at position 773; *M. bovis* BCG type exhibited three copies of a 77-bp repeat unit in addition to an A/G SNP at position 773. *M. caprae* exhibited three copies of a 77-bp repeat unit, a 34-bp deletion and one 53-bp repeat unit. *M. canettii* exhibited one copy of a 77-bp repeat unit and three SNPs following the tandem repeat in addition to a 53-bp repeat unit. ETR-D sequences of MTC type strains were deposited in GenBank under the following accession numbers (GenBank: EU180228-EU180234). ETR-D sequencing identified 98/110 MTC clinical isolates as *M. tuberculosis* including 45 isolates presenting allele 1, 26 isolates presenting allele 2, and 27 isolates presenting allele 3; the ETR-D allele did not correlate with Beijing genotype (*P* = 0.2). 5/110 isolates were identified as *M. bovis* BCG type, 5/110 isolates as *M. canettii*, and 2/110 isolates as *M. africanum* type I. All unique ETR-D sequences were deposited into our freely available database at http://ifr48.timone.univ-mrs.fr/MST_Mtuberculosis/mst.

### Comparison between ETR-D identification and reference phenotypic and molecular identifications of clinical isolates

ETR-D identification was in agreement with phenotypic identification in 107/110 (97.27%) of clinical isolates. Three isolates phenotypically identified as *M. tuberculosis* were identified by ETR-D sequencing and reference molecular methods as *M. bovis* BCG type in two cases and *M. africanum* type I in one case. Reference molecular identification agreed with ETR-D identification in 100% (110/110) of clinical isolates.

## Discussion

Previous methods for MTC species identification either combined the amplification of several genomic regions in order to identify all species [27],[48] or analyzed one gene polymorphism to distinguish between only two species. ETR-D spacer sequencing herein developed proved to be specific for the MTC and allowed the differentiation of the 7/8 MTC species in a single reaction. Indeed all the non-tuberculosis mycobacteria yielded a negative ETR-D PCR amplification as previously described [49].

The fact that *M. africanum* type II was not included in the present study may not modify this conclusion. In fact, the taxonomic status of *M. africanum* type II has been disputed [50], but it is now regarded as a phenotypic variant of *M. tuberculosis* (genotype Uganda) [51],[52]. ETR-D sequencing agreed in all cases with reference molecular identification. In this study, new mutations were identified because some genes were sequenced for the first time in some MTC species including the *oxyR* gene and MPTR in *M. canettii* and the *hsp65* gene in *M. africanum* type I (Table 3). ETR-D sequencing revealed that 3/110 clinical isolates identified as *M. tuberculosis* by phenotypic tests comprised two *M. bovis* BCG type isolate and one *M*. *africanum* type I isolate. The 497-959-bp size of ETR-D allows one-step sequencing using a modern capillary sequencer and software and may be easily sequenced using Pyrosequencing and additional internal primers. Cost was decreased in comparison with the current polyphasic approach and any microbiologist could compare the ETR-D sequence with those that we deposited in the versatile, freely accessible databank at http://ifr48.timone.univ-mrs.fr/MST_MTuberculosis/mst. This identification technique, based on PCR amplification, could be directly applied to clinical specimens exhibiting acid-fast bacilli.

ETR-D sequence identification relied not only on the variation in the number of tandem repeats illustrated by various PCR product sizes, as previously described [39] for *M. tuberculosis*, *M. africanum*, *M. bovis* group [41], but also on specific SNPs, which are stable events [53] accounting for 55.5% of genetic events observed in this study and on insertion/deletion events (accounting for 22.2% of genetic events). However, the ETR-D sequence was not correlated with the Beijing genotype as defined by Rv0927c-pstS3 intergenic region sequencing. This indicates that, although 3 ETR-D genotypes were found among *M. tuberculosis* isolates in this study, ETR-D sequencing alone cannot be used for genotyping. It is not surprising that the same, limited genomic region does not have the potential to identify at the species and strain levels. ETR-D sequencing provides, for the first time, a unique sequencing test capable of distinguishing all MTC species in a single step.

Accurate identification of MTC isolates at the species level is of particular interest in Africa where species other than *M. tuberculosis* were characterized in human tuberculosis and *M. bovis* remains a huge problem for cattle [21] (Figure 2). Their identification may direct specific epidemiological investigation. In Africa, the prevalence of *M. bovis* in human tuberculosis was correlated with the prevalence in the local cattle population [54]. Consumption of unpasteurised milk and of poorly heat-treated meat, and close contact with infected animals represent the main sources of infection for humans [3]. However, human to human transmission of *M. bovis* was recently reported in a 6-case cluster including one death due to *M. bovis* meningitis in United-Kingdom [17]. In addition, the emergence of MDR *M. bovis* has been documented, raising infection control in health care settings [55],[56]. *M. bovis* BCG type derived from the closely related virulent *M. bovis* after 230 serial passages had led to a considerably increased rate of disseminated BCG disease in HIV-infected infants reported in South Africa [57], although diagnoses were based on a few biochemical tests including the urease test and RD1deletion [58]. ETR-D sequencing allows unambiguous distinguishing of BCG type strains from *M. bovis* strains using a minute quantity of starting material. *M. africanum* identification indicated a tuberculosis microepidemic in a defined area when repeated isolation was observed [59]. Sporadic isolation of *M. africanum* strains has been reported in Europe and the United States, including outbreaks of multidrug-resistant (MDR) strains [60],[61]. In recent studies, variations in the reported prevalence of *M. africanum* among various African countries may also reflect difficulties in accurate identification of this species (Figure 2). *M. microti*, *M. pinnipedii*, *M. caprae* and *M. canettii* remain difficult to identify because of the extremely slow growth of these organisms, the difficulties with their identification under traditional bacteriological methods [62] and the fact that these recently described species have not been incorporated into current molecular identification schemes.

ETR-D spacer sequencing offers a new tool for the rapid and accurate identification of all MTC species in a single sequencing reaction without the need for expensive, time-consuming and potentially harmful polyphasic approaches. Its use could assist public health interventions and aid in the control of zoonotic transmission in African countries. Accurate identification of MTC isolates from Africa and tropical Asia would be of particular interest from the perspective of the current emergence of multidrug resistant and extended resistance isolates in these countries [63].
