# Correction: A Single-Step Sequencing Method for the Identification of *Mycobacterium tuberculosis* Complex Species

**DOI:** 10.1371/journal.pntd.0012548

**Published:** 2024-10-01

**Authors:** The PLOS Neglected Tropical Diseases Editors

The *PLOS Neglected Tropical Diseases* Editors issue this notice to resolve the concerns underlying the previously published Expression of Concern on this article [1, 2].

This study reports approval from the Institut Fédératif de Recherche (IFR) 48, Marseilles, France, but does not report approval number or date. The Expression of Concern [2] was issued due to concerns about several approvals issued by this institution.

A representative from the Aix-Marseille Université stated that the institutional investigation into the ethics concerns concluded this article meets ethical standards. They confirmed that the strains used in this study were either purchased or retrieved from clinical isolates at microbiology laboratories in France and Djibouti, as was reported in the article [1]. They also stated that no patients were included in this study, and that the study was approved by the IFR 48 ethics committee under approval #07–002 and did not require approval from a Comité de Protection des Personnes according to French law.

PLOS received a copy of the ethics approval document for #07–002: it was issued on 15 January 2007 and provides a favorable opinion for a study titled “*Identification moléculaire et génotypage des bactéries du complexe Mycobacterium tuberculosis par <<Multispacer Sequence Typing>>*”. The document specifies approval for the use of *Mycobacterium tuberculosis* isolates derived from clinical samples.

The ethics statement in the final sentence of the ‘Bacterial isolates’ section of the Methods of this article [1] is updated to:

This study was approved by the ethics committee of the Institut Fédératif de Recherche 48, Marseilles, France (approval number 07–002 issued on 15 January 2007).

With this update, the *PLOS Neglected Tropical Diseases* Editors consider the ethics approval concerns resolved.

This Correction supersedes the prior Expression of Concern [2].

Note: PLOS identified potential competing interests between the IFR 48 ethics committee that granted the ethics approval and one or more of the article’s authors.
